# Re-focusing HIV prevention messages: a qualitative study in rural Uganda

**DOI:** 10.1186/s12981-016-0123-x

**Published:** 2016-11-11

**Authors:** Sanyukta Mathur, Dina Romo, Mariko Rasmussen, Neema Nakyanjo, Fred Nalugoda, John S. Santelli

**Affiliations:** 1 Heilbrunn Department of Population and Family Health, Mailman School of Public Health, Columbia of University, New York City, 10032 USA; 2 Division of Child and Adolescent Health, Columbia University Medical Center, New York City, 10032 USA; 3 Rakai Health Sciences Program, P.O. Box 49, Entebbe, Uganda

**Keywords:** HIV prevention, Community perspectives, Risk reduction, Gender, Behavioral disinhibition

## Abstract

**Background:**

After 30 years, the human immunodeficiency virus (HIV) remains an epidemic of global concern. To support the increasing emphasis on biomedical interventions for prevention requires a renewed and reframed focus on HIV prevention messages to motivate engagement in risk-reduction activities. This paper examines youth and adult perceptions of HIV prevention messages and HIV risk assessment in a generalized HIV epidemic context in Uganda.

**Methods:**

We conducted 24 focus group discussions and 24 in-depth interviews with 15–45 year olds (n = 218) from three communities in the Rakai district of Uganda in 2012.

**Results:**

We found generational differences in the how people viewed HIV, skepticism around introduction of new interventions, continued misconceptions and fears about condoms, and gender differences in content and salience of HIV prevention messages.

**Conclusions:**

Shifts in HIV education are needed to address gaps in HIV messaging to foster engagement in risk reduction strategies and adoption of newer biomedical approaches to HIV prevention.

## Background

After 30 years, the human immunodeficiency virus (HIV) remains an epidemic of global concern; with sub-Saharan Africa bearing the heaviest burden [1]. The nature of the HIV epidemic is changing; while HIV infections among adults and children have declined in recent decades, the rate of *new* infections among adults (aged 15+ years) has stalled over the last 5 years [2]. There is considerable interest in the potential impact of biomedical approaches, such as pre- and post-exposure prophylactic HIV treatments, in reducing the burden of HIV and AIDS globally. At the same time, there are calls for concerted efforts to combine behavioral and biomedical interventions to engage at-risk populations in strategies to reduce new HIV infections [3]. Little is known about the perceived salience and continued influence of HIV prevention messages among community members within this evolving HIV context.

Uganda, located in east Africa, has a mature and generalized HIV epidemic. It has traditionally been heralded as a model sub-Saharan Africa county for curbing the HIV epidemic—from 35% prevalence in 1992 [3–5], to a national HIV prevalence of 6.4% by 2005 [4]. The early success of Uganda in reducing HIV prevalence has been engendered impassioned debates, and has been attributed in-part to an early national policy of open discussion about AIDS and messages that emphasized the risks and reality of AIDS and communications that focused on partner reduction outside of long-term marital and cohabiting partnerships (referred to as “zero grazing”) [4, 6]. It is hypothesized that these in turn triggered risk avoidance and changes in sexual behavior [5]. In the 2000s, Uganda communication strategy shifted to the promotion of abstinence among unmarried youth [5] and condom use with casual sexual partners [4]; along with an emphasis on voluntary HIV counseling and testing (VCT) [3, 7–10]. In the last decade Uganda has been at the forefront of HIV treatment programs; providing prevention of mother-to-child transmission services and anti-retroviral treatments (ARTs) to many HIV-infected people [11, 12]. A recent analysis of print media coverage on HIV and AIDS in Uganda after 2003 found increased reporting on new biomedical interventions and treatments for HIV, rather than earlier strategies aimed at HIV risk and behavior change [13].

In recent years however, Uganda has experienced an increase in HIV prevalence nationally; the most recent national sero-behavioral survey indicated an increased HIV prevalence of 7.2% among 15–49 year olds in Uganda [12, 14]. These recent increases in HIV prevalence have reinvigorated an interest in the HIV prevention messages and programs that are currently being implemented, and how they are being understood by community members. In particular, there is concern about behavioral disinhibition because HIV messages in country may now emphasize HIV testing and treatment, rather than sexual behavior change strategies [15, 16]. This study aimed to elucidate and examine gendered and generational perspectives on HIV prevention messages in Uganda.

Exploration into perceptions of HIV messages can provide important insights into how HIV messages are perceived and understood at the community-level. These insights can provide useful input into reframing or refocusing of HIV prevention campaigns, programs and policies in high HIV prevalence contexts. We used data from focus group discussions and individual in-depth interviews conducted with adolescent and adult respondents in rural Uganda to examine community perceptions of HIV prevention messages. Understanding the salience of HIV messages from a community perspective can guide the refinement of strategies to enhance engagement in risk-reduction activities and help create more effective HIV prevention efforts.

## Methods

### Study site

Uganda has a population of over 32 million, with a relative young age structure (median age is 15 years) and fairly low life expectancy at birth (52.7 years) [17]. The majority of the population lives in rural areas and relies on agriculture and subsistence farming. Educational attainment is low overall, and disparities persist by age, gender, and area of residence. For example, men are more likely to be educated compared to women; the median years of schooling completed by men is 5.7 years, while for women it is 5.2 years [18]. People living in rural areas are less educated than those in urban areas. And younger generations are more likely to have attended school compared to older generations. Similar patterns exist in regards to exposure to mass media (newspapers, TV, radio). Over 24% of women in rural areas say they do not access any type of mass media on a weekly basis, compared to only about 13% of men [18].

This study was part of a 5-year research initiative to examine behavioral, biological, and demographic risk and protective factors associated with incident HIV infections among youth in rural south-central Uganda [19–23]. One of the aims of the study was to assess how HIV risk perceptions and understanding of risk reduction strategies has changed over time. The data collection was conducted with community members who are part of the Rakai Health Sciences Program (RHSP) and its longitudinal epidemiological surveillance cohort. The cohort includes 15–49 year olds selected from 33 communities within the Rakai district interviewed in surveys conducted every 12–20 months [24]. In the cohort, the average age of the respondents is 28 years for men and 27 years for women. Educational attainment in cohort is reflective of the national statistics, only a third of the cohort participants have completed any secondary school. Most of the respondents (more than 80%) live in rural communities. Forty percent of women and 29% of men think they are at risk of HIV acquisition, while 78% of men and women report that they don’t know the HIV status of at least one of their partners [25]. Men report having more sexual partners compared to women, and reported consistent condom use is low. The community HIV prevalence in this cohort is 11% [25].

### Data collection

The data for this analysis rely on 24 focus group discussions (FGDs) and 24 individual in-depth interviews (IDI) conducted in 2012 with a total of 218 respondents aged 15–45 years, sampled from two rural communities and one peri-urban community in the southwestern district of Rakai, Uganda. Using proportionate sampling communities were randomly selected from within the Rakai cohort. Group discussions were chosen as an apt method to elicit group perceptions and collective recall around HIV prevention messages. Additional IDIs were conducted to allow for an exploration of individuals’ interpretation and response to HIV prevention messages. Two FGDs (one with males and one with females) were conducted in each of the three communities with four age groups—current teenagers (15–19 year olds), young adults (20–24 year olds), adults (25–34 year olds), and older adults (35–45 year olds) for a total of 24 FGDs. The four age-group generations were defined to capture respondents who had experienced different generations of HIV/AIDS messages and programs in Uganda. FGD and IDI participants were randomly select from the list of Rakai cohort participants to by age and sex. Each group discussion included nine participants, on average. IDIs were conducted with eight participants in each of the three communities representing the same age groups as in the group discussions.

### Procedures

Semi-structured field guides were used to facilitate the FGDs. The guide focused on (1) general attitudes about HIV and AIDS in the community, (2) HIV prevention or risk-reduction messages that respondents received as youth and as adults, and the source of these messages, and (3) larger social policies (e.g. implementation of universal primary education) that might have influenced sexual risk behaviors. When probing about HIV-related messages, we inquired specifically about perceived impact or behavior changes that they and their peers had made in response to the messages. In this analysis, we focus on the HIV-related messages and their perceived impact.

Semi-structured field guides were also used to guide IDIs. Participants were recruited randomly from a list of Rakai participants. Interview questions focused on HIV prevention messages received during key life events or stages (such as, puberty, marriage, migration, or pregnancy). For each life stage mentioned, respondents were probed on the most prominent HIV prevention message received and specifically how this HIV message affected their HIV risk perception, behavior change or services accessed. Participants were also asked about specific public HIV/AIDS messages throughout this region such as drums calling for HIV prevention, love carefully/love faithfully, ABC (Abstinence, Be faithful, use Condoms), zero grazing, cross-generational sex, and get off the sexual network. If answered in the affirmative, participants were asked to relate these public messages to the main life-events that they had listed.

FGDs were conducted in easily accessible community locations, such as local health centers, while IDIs were conducted in a private setting at the participant’s home or place of work. RHSP qualitative research team, including four men and four women in the 20–40 age range and representing various religious and ethnic groups local to the south western region of Uganda conducted the FGDs and IDIs. Male interviewers conducted discussions with male respondents, as did the females. The RHSP research team had previous experience in collecting data on HIV and related topics in this area. All data collection was conducted in the local language, Luganda, by the RHSP qualitative research team; interviews were tape-recoded, transcribed and translated from Luganda to English. The records for each FGD and IDI included verbatim transcripts and summary notes from the discussion facilitators/interviewers.

### Analysis

We employed a systematic analysis of narrative data, which involved both within-case and across-case analysis [26, 27]. The first, second, and third author developed a central list of codes of themes emerging from a close analysis of the data; this was done separately for FGDs and IDIs separately [28]. Each transcript was coded by either the second or third author using the appropriate list. Once the data were coded, the authors sorted the coded data into tables for each key theme. These tables/matrices allowed cross-referencing and comparison of statements by male and female respondents and by age group. The matrices used for FGD and IDI data analysis included information on the messages that respondents received and their perceptions of the impact of specific messages and HIV services. Codes from the individual transcripts were also applied to a similar matrix. The authors then reviewed the matrices to look for patterns in the data, identify points of convergence and divergence, and summarize findings by gender and age group.

### Ethics, consent and permissions

All participants provided informed consent prior to participation in the study. Each participant was compensated for their time and reimbursed for transportation costs, per local ethical guidelines. All participants were interviewed in private locations, away from onlookers. To ensure confidentiality, no personally identifying information (such as address or phone number) was retained in the qualitative transcripts. The presentation presents either aggregate data or identifies respondents by generic respondent categories. Institutional review board (IRB) approvals for this data collection and analysis were obtained from the Science and Ethics Committee of the Uganda Virus Research Institute and the National Council for Science and Technology and from the IRBs at Columbia University Medical Center and Johns Hopkins University.

## Results and discussion

**Fig. 1:**
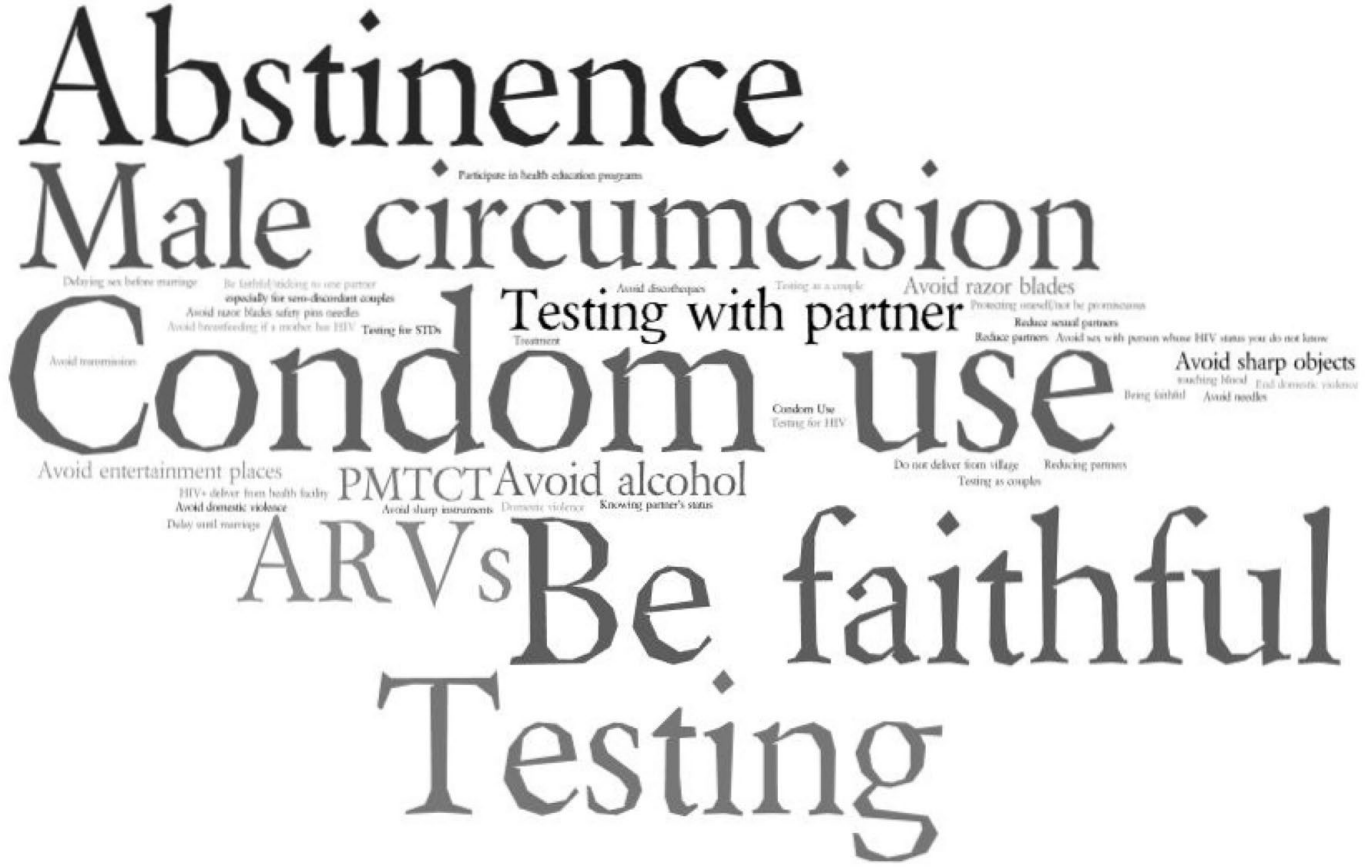
Prominent HIV and AIDS messages in Rakai, Uganda

Messages related to HIV/AIDS were ubiquitous in the Rakai communities. When respondents were asked to list and rank the main HIV prevention messages they had heard or received throughout their lifetimes, we found that respondents, regardless of age or community of residence, had been reached by a variety of HIV prevention and risk-reduction messages through a variety of mediums ranging from mass media campaigns to community health events, and through interpersonal interactions with health care workers. We did not find any major differences in the perceptions of HIV messages in the rural and peri-urban communities in Rakai, and thus present date by age and gender instead. Figure 1, presents a visualization of the wide variety of HIV messages reported by respondents. Using the data from the listing and ranking exercise, messages noted with higher frequency are presented in larger font while the messages that were noted less frequently are noted in the smaller fonts. The image presents a mix of prominent HIV prevention messages, and community perceptions of prevention strategies to avoid risk. For instance, respondents across age, gender, and site noted receiving messages on being abstinent, being faithful, and using condoms (i.e., ABC messages)—messages that have been emphasized in the Ugandan HIV prevention campaigns. Respondents also noted more recent HIV messages around HIV testing, medical male circumcision, and the use of anti-retrovirals. At the same time, messages to avoid sharp objects and razor blades to avoid blood contact with an infected individuals or avoiding alcohol or entertainment places (e.g. nightclubs, bars, etc.), were also noted. These messages, reflected the respondents’ and community perceptions around strategies to avoid spaces and activities associated with HIV risk.

### 1. Generational Perspectives on Fear of HIV

All respondents felt that HIV and AIDS remained a major health concern in the community. The older generation feared HIV as a result of their personal experiences with HIV (i.e., family members dying of HIV) which they noted drove them to adhere to preventive behaviors. The majority of older participants (ages 35+) described several accounts of witnessing death and severe illness as a result of HIV. Older respondents, noted that their experiences of hardship, loss, and suffering due to AIDS, were often times the impetus for changing behavior such as remaining faithful to one partner, abstaining from sexual activities if not in a committed relationship, and seeking condoms or HIV testing services.

The younger respondents in our study however expressed clear motivations for HIV prevention because of their heightened awareness of risk due to HIV health education messages. Though older respondents believed that young people no longer feared HIV given its lower impact in the community compared to 15–20 years ago; younger respondents often noted adapting preventive behaviors after recognizing their behaviors as risky, usually as a result of hearing HIV prevention messages. Several respondents noted that they adjusted their perception of HIV risk, based on HIV messages. For instance, one 25 year old young man felt he may be at risk for HIV and other STIs since he was unsure whether his partner had other sexual partners. Fear of HIV also prompted many young men to seek out voluntary medical male circumcision (VMCC) as a prevention method. For example, a 23 year old young man noted that he decided to go for circumcision when he received education that “if you are circumcised you stand more chances of not getting the HIV infection.”

### 2. Skepticism around the introduction of new technologies—condoms and HIV treatment

There were also interesting parallels in community perceptions with the introduction of condoms and HIV treatment in the Rakai community. Twenty years ago, HIV/AIDS and condoms were new in Rakai and there was skepticism about condoms and fear that they could lead to behavioral disinhibition. Older respondents noted that, when first introduced for HIV prevention, condoms were believed to be the cause of HIV. One group of 35–45 year old men stated, “Most of our friends feared this [condoms]. They used to say that condoms contained HIV.” Respondents also noted that early in the epidemic some religious leaders denounced condoms on the grounds that they promote promiscuity. In the past, substantial fear had co-existed with limited knowledge and scant availability of condoms.

In an interesting parallel, respondents were equally skeptical of antiretroviral therapies (ARTs). ARTs first became available in Rakai in 2004, with scaled-up outreach by 2005, nearly 87% of the community knew where to access treatment [29]. Respondents universally acknowledged the improvement in quality of life and reduction of suffering for people living with AIDS due to HIV treatments. At the same time, during the discussions and interviews, we found that the respondents did not grasp the potential of ARTs in reducing HIV viral loads (or infectiousness of the infected individual) leading to reduced HIV transmission. Instead, respondents stated that ARTs had hidden the HIV epidemic and with treatment it was no longer possible to tell who was HIV-positive.

ARTs have helped us but this has come along with a challenge. When people use ARTs all the signs [of AIDS] on their bodies disappears and [they] spread the infection to others who may not be in position to tell if that person is on drugs. (Males, 20–24 year olds, FGD)

This hidden epidemic, and the inability to identify and avoid HIV-positive individuals, respondents felt further fueled the epidemic. These sentiments appeared often from group discussions with younger respondents, as shown in the quotes below.

HIV has increased [in the community] because if an HIV positive person starts on drugs before showing any signs of HIV/AIDS it is hard to differentiate him/her from that one who does not have HIV. And if the young man is the one who has HIV, he makes sure that he infects other people because there is nothing [no sign] which shows that he is sick [has HIV]. (Females, 20–24 year olds, FGD)

P1: After introduction of drugs (ARVs) people get them and stay longer.

P6: But this has also brought more problems because people using these drugs look so healthy. People end up indulging in sex with them and get infected as well. (Males, 15–19 year olds, FGD)

Similar to earlier behavioral disinhibition related fears about condoms, respondents also felt that because of ARTs, AIDS might no longer be seen as a debilitating illness. In turn, respondents felt that this removal of the fear of AIDS led to the abandonment of protective behaviors among HIV-positive individuals, such as the use of condoms or limiting the number of sexual partners.

When people use ARTs they regain normal health and look nice. This compels them to behave recklessly because they now feel well. Remember this person is already infected. They do not mind about any form of protection, they adopt promiscuous behaviors. (Males, 20–24 year olds, FGD)

These ideas were particularly prevalent in the group discussions with younger respondents. A group of 15–19 year old men reiterated this idea of behavioral disinhibition and noted, “Some young men have decided to have sex with each and every girl they come across. He does this because he knows there is ‘septrin’ and ‘ARTs’.”

### 3. Continued misconceptions and fears about condoms

Discourse on condoms focused almost exclusively on adolescents, particularly unmarried adolescents. Adult respondents did not feel that the messages around condoms were targeted toward them, but toward youth instead. Adult respondents noted that many youth did not use condoms despite their ready access and known effectiveness. A 37 year old teacher noted,

“You find some young boys carrying condoms... When we sit with them in their small groups they tell you condoms are not all that effective. That is what they tell you when you talk about condoms. This is an indicator that they do not use condoms. This is dangerous."

Younger respondents revealed surprising misconceptions about how condoms work. Several younger respondents noted fear or anxiety that condoms or part of condoms could get lost inside women. Several linked condom use to cervical cancer or sterility, as demonstrated by the quote below.

Some young women don’t want to use condoms because some say that condoms cause cervical cancer and so she may see that she has not yet produced a child, and by the time she wants to produce, the uterus will have been removed. So, most young women don’t want to use condoms. (Females, 20–24 year olds, FGD)

Another common theme among the youth was the sense of shame or embarrassment in acquiring or carrying condoms. A young man noted, “They [condoms] were there at the shops but feared to go and buy them.” Another young man, reflecting on his teen years noted being unable to get condoms for fear of being questioned by the shopkeepers about his sexual activity:

No, I did not use a condom. I used to fear such things. I could not go to a shop to ask for a condom. They would question me. (Male, age 23, IDI)

These misconceptions and social barriers for condom acquisition are particularly surprising given the depth of HIV education in this region and the focus on condom use for risk reduction.

### 4. Gender differences in content and salience of HIV messages received

Although factual information about HIV transmission and prevention strategies is not intended to vary widely between males and females of different age groups, we found that males and females across generations reported receiving messages that emphasized different types of prevention behaviors. Women in all age groups (15–45 years) reported receiving more messages related to individual behavior change—abstinence, being faithful to their partner—and received them consistently throughout their lifetime. Men were less likely to report receiving HIV prevention messages around abstinence, being faithful, and partner reduction. Few women reported receiving messages promoting (or emphasizing negotiation of) condom use. Adolescents and younger men, however, reported an emphasis on service-based messages about condom use and, more recently, male circumcision. The source of these messages also varied between males and females. Women noted that they received more HIV/AIDS education throughout their lifetime and received it more directly through interpersonal interactions from health providers, teachers, and parents. Instead, the male respondents noted receiving most HIV messages through public or less interpersonal sources such as the radio or during class time.

Women reported that HIV prevention messages targeting faithfulness to one’s sexual partner were messages that they received throughout their life. This was also the primary HIV prevention message that women felt was salient to them. Female respondents spoke at length about being faithful to their partners to lower their risk of HIV acquisition. At the same time, several of the women who relied on this strategy acknowledged that their partners did not adhere to this strategy, as exhibited by the quote below.

Respondent: I wanted to remain with my husband and I did not want to get HIV/AIDS. So, I did not want to have any other sexual partner.

Interviewer: Did your husband behave the same way like you during that time? How did he behave?

Respondent: He had another woman.

(Female, age 22, IDI)

The effectiveness of the faithfulness HIV prevention message is clearly dependent on the actions of both partners. However, in both group discussions and interviews, women discounted men’s ability to refrain from multiple or concurrent sexual partnerships, even while they themselves adopted partner reduction as an HIV prevention strategy.

The women have managed to protect themselves, but the men are promiscuous. For example, you can stop a man from loving a certain woman but tomorrow he will be with another woman…Men who are faithful to their wives are few.

(Females, 25–34 year olds, FGD)

Interviewer: Did your husband behave the same way [being faithful to husband] as you?

Respondent: Do you think men can be faithful to their wives? Not at all!

(Female, age 30, IDI)

## Conclusion

In this analysis we set out to understand community members’ perceptions of HIV risk reduction messages. Our analysis highlights these perceptions within the context of a long-standing HIV program. Despite concerted health education efforts over the last three decades in Rakai, there are gaps in health information, ways the messages are used, and their salience. If ignored, these gaps could hamper future HIV behavioral and biomedical prevention efforts.

Previous research has highlighted the importance of tailoring HIV education efforts according to a variety of contexts and individual practices, such as cultural and religious beliefs, gender, socio-economic status, and sexuality. However, we found important gendered differences in the content, sources, and frequency of HIV-prevention messages received by men and women, potentially leading to ineffective application of these strategies. While men are an appropriate target for condom and male circumcision messages, these strategies should also be impressed upon women in order to improve the adoption and effectiveness of these prevention methods across the general population. Abstinence and messages related to being faithful to their partners should also be emphasized for men. Other research suggests that recent HIV prevention efforts in Uganda have de-emphasize partner reduction strategies [15, 16]. We found that the partner reduction message was common, but was being used asymmetrically. Women are receiving messages about being faithful to their partners and are adhering to them even when they know that their partners are engaged in extra-marital partnerships. Perhaps this is one HIV prevention strategy that women feel they efficaciously apply, however without their partner’s participation, women open themselves up to HIV risk through their partners’ extended sexual networks. This message thus, is less likely to be successful at reducing HIV risk if not adhered to by both partners. HIV prevention efforts need to acknowledge and address broad based gender differentials in how HIV preventive messages and services are discussed with men and women throughout their life cycle.

We also found that respondents blamed HIV treatment and prevention methods for increasing the spread of HIV, in contrast to global discussions about the promise of HIV *treatment as prevention* approach [30, 31]. Even though, ARTs had been available in the study site for over a decade at the time of the research, in our sample, neither younger nor older respondents understood the potential of ARTs to prevent HIV transmission. Instead ARTs were assumed to promote behavioral disinhibition. Other research in the region also has found that ART availability is often perceived to increase the spread of HIV [32, 33]. Rakai residents did not understand how HIV treatments could reduce transmission and many respondents erroneously believed that ART accelerated and perpetuated HIV transmission.

Participants in our study were concerned about behavioral disinhibition, particularly among youth, due to the availability of HIV prevention and treatment methods. While it is true that young people in Rakai now often do not have the same personal experiences with HIV/AIDS, we found that youth are aware of their risk and seemed motivated to engage in preventive behaviors. Previous literature from Uganda shows improved sexual and reproductive health knowledge, behaviors, and access to services among young people aged 15–24 between 2003–2004 and 2012 [34]. In fact, research in Rakai has found dramatic declines in sexual risk behaviors among youth between 1999 and 2011 [22]. Thus, there might be a disconnect between fear of behavioral disinhibition and evidence for that—at least among youth in Rakai. Further, another recent analysis from Rakai found that declines structural interventions, like universal primary schooling, might be contributing to reduced HIV and pregnancy risk among young people [35].

There is extensive literature about the ongoing misconceptions about condoms and the reluctance to use them. Indeed within the RHSP cohort, consistent condom use remains low [25]. We were surprised to find that despite decades of intensive HIV/AIDS education in Rakai condom use among youth continued to be confounded by misconceptions and shame—aspects which might be contributing in part to their limited uptake. Previous research also had demonstrated challenges ranging from limited access, to generational and gendered power dynamics that make it difficult to negotiate condom use, among youth [36, 37]. Sustained efforts are needed to increase in-depth knowledge, dispel myths, and enhance access to condoms and HIV-prevention services for youth.

## Limitations

Our analyses are based on the subjective responses of Rakai community members. The interviewers were part of the extended RHSP team; RHSP has been providing HIV education and services to these communities for an extended period of time. Thus, it is possible that respondents were providing socially desirable responses. These biases might be particularly relevant in the discussion around the influence of messages on behaviors. However, the consistency of responses from focus group and in-depth interview discussions increase our reliability and suggest the pervasiveness of the themes presented here.

## Implications

While global discourse on HIV prevention is emphasizing biomedical approaches (medical male circumcision, treatment as prevention, pre and post exposure prophylactics) for HIV prevention, these approaches do not discount the need for continued behavioral prevention efforts through health education to encourage behavior change [38]. In fact, biomedical approaches can fail if the underlying social and behavioral motivations are not well understood or addressed. Further, HIV prevention efforts need to include a wide range of social and structural interventions that might facilitate uptake of preventative behaviors. In this paper we highlight needed shifts in the HIV education and prevention strategies to address sexual double standards related to partner reduction strategies, to educate the public on treatment as prevention, and to dispel myths related to behavioral disinhibition. In this third decade of the HIV epidemic, we need to provide nuanced information and re-posit strategies to enhance engagement in HIV risk-reduction activities.

## Abbreviations

ABC: abstinence, be faithful, use condoms
ART: anti-retroviral treatments
FGD: focus group discussions
HIV: human immunodeficiency virus
IDI: in-depth interviews
IRB: institutional review board
VCT: voluntary HIV counseling and testing
